# Risk Stratification in Primary Gastric Malignancies in Children, Adolescents, and Young Adults: A SEER-Based Analysis

**DOI:** 10.3390/jpm16040198

**Published:** 2026-04-01

**Authors:** Stavros P. Papadakos, Ioannis Katsaros, Stamatina Vogli, Alexandra Argyrou, Ioannis Karniadakis, Ioannis A. Ziogas, Paraskevas Gkolfakis, Stamatios Theocharis, Dimitrios Schizas

**Affiliations:** 1 First Department of Gastroenterology, Medical School, National and Kapodistrian University of Athens, Laikon General Hospital, 11527 Athens, Greece; stavrospapadakos@gmail.com (S.P.P.); argyalex89@gmail.com (A.A.); 2 First Department of Pathology, Medical School of National and Kapodistrian University of Athens, Laikon General Hospital, 17 Agiou Thoma Str., 11527 Athens, Greece; 3 First Department of Surgery, National and Kapodistrian University of Athens, Laikon General Hospital, 17 Agiou Thoma Str., 11527 Athens, Greece; gikats.md@gmail.com; 4 Department of Gastroenterology, “Metaxa” Cancer Hospital, Botasi 51, 18537 Piraeus, Greece; stamvog95@gmail.com; 5 Upper Gastrointestinal Surgery, Department of General Surgery, St. George’s Hospital, St. George’s University Hospitals NHS Foundation Trust, London 84790, UK; ioanniskarniadakis@gmail.com; 6 Department of Surgery, Medical Campus, University of Colorado Anschutz, Aurora, CO 80045, USA; ioannis.ziogas@cuanschutz.edu; 7 Second Department of Gastroenterology, Medical School of National and Kapodistrian University of Athens, University General Hospital “Attikon”, 11527 Athens, Greece; pgolfakis@gmail.com

**Keywords:** paediatric gastric cancer, young adults, adenocarcinoma, signet ring cell carcinoma, survival analysis, SEER database

## Abstract

**Backgrounds:** Gastric malignancies are exceptionally rare among children and young adults, and their clinicopathological characteristics and outcomes remain poorly defined. This study aimed to evaluate the demographic, clinical, and survival features of gastric cancer in patients aged ≤24 years using population-based data. **Methods:** Data were extracted from the Surveillance, Epidemiology, and End Results (SEER) database (2004–2016) for individuals ≤ 24 years with primary gastric malignancies. Cox proportional hazards models were used to identify predictors of overall survival (OS). **Results:** A total of 324 patients were included (mean age: 20 years; 50% female). Adenocarcinoma was the most common histologic subtype (52%), nearly half of which were signet ring cell carcinomas (46%). The majority of patients (59%) were diagnosed at stage IV. Median OS was 18 months, and 5-year OS was 49%. Children and adolescents (≤18 years) had significantly better survival than young adults (median OS: 34 vs. 15 months, *p* < 0.01). In multivariable analysis, advanced AJCC stage and higher lymph node ratio were independently associated with worse overall survival, while lymphoma histology and radiotherapy were linked to more favourable outcomes. **Conclusions:** Gastric cancers in patients ≤ 24 years often present at advanced stages and are dominated by aggressive histology. Prognosis is primarily determined by tumour stage, histological subtype, and lymph nodal burden. These population-based risk estimates support risk stratification and prognostic assessment in young patients by highlighting histology- and nodal-burden-driven prognostic strata.

## 1. Introduction

Paediatric cancer affected 9620 children (aged 0–14 years) and 5290 adolescents (aged 15–19 years) in 2024, resulting in an estimated 1040 and 550 deaths in the United States, respectively [1]. Although young adults account for only about 5% of all gastric cancer cases, there has been a reported rising incidence, particularly among white adults between 25 and 39 years [2]. Gastric tumours are relatively rare in both children and adolescents, accounting for only a small fraction of cases [3,4]. The most frequent clinical manifestations of gastric carcinoma in paediatric patients include abdominal pain, followed by vomiting, anaemia, and unintentional weight loss [5].

Due to its rarity, the clinicopathological features and outcomes of gastric malignancies in young patients remain poorly characterised [6,7,8]. Due to the aggressive nature of gastric cancer in young patients, particularly those aged 40 years or younger, the disease often presents with advanced clinicopathological features, including a higher prevalence of diffuse-type adenocarcinoma, signet ring cell histology, and poor differentiation, as observed in studies across European, U.S., and Latin American populations [6,7,8]. Young patients, especially females, are more likely to be diagnosed with metastatic disease (59–79%) compared to older patients, with a higher incidence of bone metastases and stage IV disease [7]. These characteristics, coupled with a predominance of women in younger cohorts (46–57%) and a significant proportion of Hispanic patients in U.S. and Latin American studies, contribute to a worse prognosis, with median overall survival for young patients with metastatic disease ranging from 5 to 8 months compared to 11 to 13 months for older patients [7,8]. Despite these challenges, survival outcomes in young patients are heavily influenced by clinical stage, treatment modality, and tumour grade, with curative resection significantly improving 5-year survival rates (up to 64% in young European adults), though non-curative resections and advanced stages (III–IV) are associated with poorer outcomes across all age groups [6].

The aim of the current study was to describe age-related patterns in clinicopathologic characteristics, management, and survival in patients ≤ 24 years with primary gastric cancers using U.S. cancer registry data.

## 2. Materials and Methods

### 2.1. Study Protocol and Data Source

This study was conducted in accordance with a protocol agreed a priori by all the authors and registered in Open Science Framework (registration number: osf.io/vgzrm). Data were retrieved from the Surveillance, Epidemiology, and End Results (SEER) 18 Registries Research Data, November 2022 submission, which covers approximately 28% of the U.S. population. The SEER program collects information on cancer incidence, patient demographics, tumour characteristics, treatment, and survival from population-based cancer registries across multiple U.S. regions. Case selection and data extraction were performed using SEER*Stat software version 8.4.1.2 (National Cancer Institute, Bethesda, MD, USA).

### 2.2. Study Population: Inclusion Criteria

We included all patients aged ≤24 years at diagnosis who were identified in the SEER database with a first primary malignant neoplasm of the stomach between 1 January 2004 and 31 December 2016. The upper age limit of 24 years was chosen because it is widely used to define the adolescent and young adult population in oncology research. For subgroup analyses, patients were stratified into three predefined age groups: children (<15 years), adolescents (15–19 years), and young adults (20–24 years), in accordance with commonly used age categories in epidemiological studies of paediatric, adolescent and young adult cancers. Gastric malignancies were identified using the International Classification of Diseases for Oncology, Third Edition (ICD-O-3) topography codes C16.0–C16.9 and behaviour code 3 (malignant). Only pathologically confirmed cases were included. Patients with missing age, follow-up time, or vital status data were excluded from the analysis.

### 2.3. Variables and Definitions

We extracted the following variables from the SEER database: age at diagnosis, sex, race (White, Black, Other), year of diagnosis (categorised into three periods: 2004–2008, 2009–2012, and 2013–2016), and tumour characteristics, including primary site, histological subtype, histological grade, AJCC stage group (I–IV), and presence of distant metastases (bone, liver, lung). Treatment variables included surgical resection (yes/no), chemotherapy (yes/no), and radiation therapy (yes/no). Chemotherapy was recorded as a binary variable (yes/no) in SEER, without information on treatment timing (neoadjuvant vs. adjuvant), intent (curative vs. palliative), or combination with surgical resection.

The lymph node ratio (LNR) was calculated by dividing the number of positive lymph nodes by the total number of examined lymph nodes. This variable was analysed both as a continuous covariate and, in sensitivity analyses, as a categorical variable based on tertiles (low, medium, high).

The primary outcome was overall survival (OS), defined as the time from diagnosis to death from any cause or last known follow-up. Survival time was calculated in months and censored at the date of last contact for patients who were alive. Secondary outcomes included cancer-specific survival (CSS), defined as the time from diagnosis to death attributed to gastric cancer, and subgroup analyses of OS according to age group, histological subtype, AJCC stage, lymph node ratio, and year of diagnosis.

### 2.4. Statistical Analysis

Descriptive statistics were used to characterise the study population. Categorical variables were summarised as frequencies and percentages, while continuous variables were described using means and standard deviations (SDs).

Univariate Cox proportional hazards regression analysis was used to estimate the association between each covariate and OS. The variables included in multivariable Cox regression were selected a priori based on biological importance and data availability to avoid the inferential limitations of selecting variables for multivariable models based on univariable comparisons or stepwise procedures [9]. The final multivariable Cox regression model included: age group, sex, year of diagnosis, race, histology, AJCC stage, treatment modalities (surgery, chemotherapy, radiation), and LNR.

To assess the robustness of the findings, four sensitivity analyses were performed: (1) a multivariate Cox regression model in which the adenocarcinoma histological subtype was stratified into signet ring cell carcinoma and other adenocarcinomas; (2) a complete-case analysis including only patients with no missing data; (3) a model treating lymph node ratio as a categorical variable based on tertiles (low, medium, high); and (4) a model excluding cases with rare histological subtypes. All sensitivity models were adjusted for the same covariates as the primary analysis.

Results were reported as hazard ratios (HRs) with 95% confidence intervals (CIs) and corresponding *p*-values. Model fit and proportional hazards assumptions were evaluated using standard diagnostics including Schoenfeld residuals. Statistical significance was set at a two-tailed *p*-value < 0.05. All analyses were performed using R version 4.4.3 with the survival, dplyr, survminer, and broom packages.

### 2.5. Ethical Considerations

This study was based on publicly available, de-identified data from the SEER program and hence does not constitute human subject research. As such, it was deemed exempt from institutional review board (IRB) review and informed consent requirements.

## 3. Results

### 3.1. Patients’ Characteristics

A total of 324 patients aged ≤24 years with primary gastric malignancies were included in the analysis. The mean age at diagnosis was 20.1 years (SD: 4.3). The cohort consisted of equal numbers of female and male patients (50.0% each). Anatomical site was available for 197 patients (61.0%). Among these, the most common tumour locations were the body of the stomach (n = 71; 36.0%), the antrum (n = 53; 26.9%), and the cardia (n = 47; 23.8%), followed by the fundus (n = 16; 8.1%) and the pylorus (n = 10; 5.2%).

Histologically, the most frequent tumour type was adenocarcinoma (n = 168; 51.9%), of which 45.8% were signet ring cell carcinomas, followed by sarcomas (n = 59; 18.2%), lymphomas (n = 56; 17.3%), neuroendocrine carcinomas (n = 27; 8.3%), and tumours of unspecified type (n = 14; 4.3%). Data on metastatic involvement were available in only 45.9% (n = 149) of cases and corresponded to documented M stage (M0/M1). Among these, information on specific metastatic sites was available only for a subset of cases, with 9 patients (6.1%) presenting with bone metastases, 25 (16.8%) with liver metastases, and 16 (10.7%) with lung metastases. For the remaining cases, metastatic status was recorded as “blank,” “unknown,” or “not applicable,” and therefore could not be reliably interpreted or included in comparative analyses. Nodal staging information (N stage) was available for 113 patients (34.8%). However, at least one lymph node was examined in 246 patients (75.9%), allowing for the calculation of lymph node ratio (LNR) in the majority of the cohort. AJCC stage was documented for 199 patients (61.4%); the majority (n = 117; 58.8%) were diagnosed at stage IV, while stages I, II, and III accounted for 26.6% (n = 53), 8.0% (n = 16), and 6.5% (n = 13), respectively. Regarding tumour grade, most neoplasms were poorly differentiated (grade III, n = 67; 39.0%), although grading was missing in nearly half of the cases (47.0%). Baseline demographic, clinical, and tumour characteristics of the cohort are summarised in Table 1.

No statistically significant differences in mean age at diagnosis were observed between sexes (female: 20.2 years, male: 20.0 years; *p* = 0.62), year of diagnosis groups (2004–2008: 20.1 years, 2009–2012: 19.7 years, 2013–2016: 20.4 years; *p* = 0.40), or racial groups (White: 20.0 years, Black: 19.9 years, Other: 20.3 years; *p* = 0.94). In contrast, a significant difference was noted between histological subtypes (*p* < 0.001). Post-hoc comparisons demonstrated that patients with lymphomas and sarcomas were significantly younger (mean age 17.5, SD 6.2 and 18.7 years, SD 4.7, respectively) at diagnosis compared to those with adenocarcinomas (mean age 21.3 years, SD 2.7).

### 3.2. Survival Outcomes and Prognostic Factors

**Figure 1:**
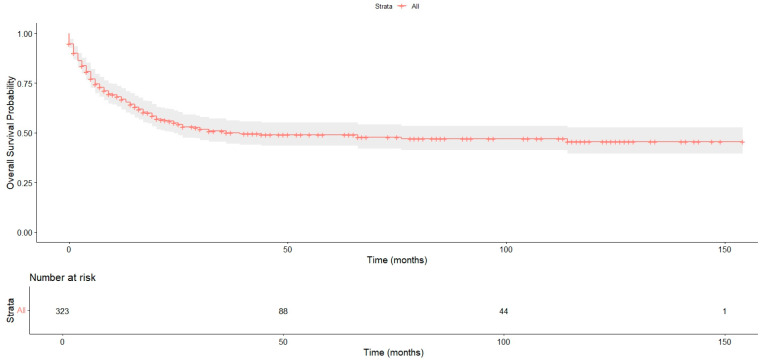
Kaplan–Meier overall survival curve for the entire cohort, illustrating a marked early decline in survival followed by a gradual plateau over time.

Median follow-up for the cohort was 49 months (IQR: 20–102 months), while the median overall survival (OS) was 18 months (IQR: 5–64 months). The 1-year and 5-year OS rates were 66.7% (95% CI, 61.6–71.2%) and 49.0% (95% CI, 43.4–55.2%), respectively. Median cancer-specific survival was 6 months (IQR: 2–16 months). The overall survival curve for the entire cohort (Figure 1) demonstrates a steep decline during the first 12–24 months following diagnosis, followed by a more gradual plateau, indicating that mortality is highest early in the disease course, particularly among patients with advanced-stage disease.

No statistically significant differences were observed in OS across year of diagnosis groups (2004–2008, 2009–2012, and 2013–2016; log-rank *p* = 0.50). In contrast, overall survival differed significantly between paediatric (≤18 years) and young adult (19–24 years) patients, favouring the younger group. Patients aged ≤18 years demonstrated substantially longer survival compared to those aged 19–24 years (median OS: 34 vs. 15 months, respectively; log-rank *p* < 0.01). Moreover, OS differed significantly across histological subtypes (log-rank *p* < 0.01). Patients with adenocarcinomas, including signet ring cell carcinoma, exhibited the poorest survival (median OS 12 months), with the signet ring cell carcinoma subtype demonstrating a median OS of only 9 months. In contrast, patients with lymphoma (67 months), neuroendocrine carcinoma (40 months), and sarcoma (44 months) had markedly more favourable outcomes.

In univariate Cox regression analysis, increasing age was significantly associated with worse overall survival. Compared to children aged 0–14 years, patients aged 20–24 years had a significantly higher risk of death (HR 3.16, 95% CI 1.54–6.47), while no statistically significant difference was observed when compared to the 15–19 years group (HR 1.95, 95% CI 0.89–4.29). Compared to AJCC stage I, the risk of death was significantly increased for stage II (HR 3.08, 95% CI 1.07–8.91), stage III (HR 8.00, 95% CI 3.07–20.86), and stage IV disease (HR 13.75, 95% CI 6.62–28.58).

In the univariate Cox regression analysis, patients with lymphoma (HR 0.14, 95% CI 0.07–0.26, *p* < 0.001), neuroendocrine carcinoma (HR 0.18, 95% CI 0.08–0.40, *p* < 0.001), and sarcoma (HR 0.14, 95% CI 0.08–0.27, *p* < 0.001) had a significantly lower risk of mortality compared with those with adenocarcinoma. The “unspecified” histology group did not differ significantly from adenocarcinoma (HR 0.89, 95% CI 0.43–1.83, *p* = 0.747). When signet ring cell carcinomas were compared with other adenocarcinoma subtypes, the hazard ratio was not significantly different [HR 1.07 (95% CI 0.74–1.54), with signet ring cell carcinoma as the reference category].

Surgical resection was associated with significantly improved survival (HR 0.32, 95% CI 0.22–0.47), while chemotherapy was associated with an increased risk of mortality (HR 2.23, 95% CI 1.56–3.19). No significant associations were found for sex, race, year of diagnosis, radiation therapy, or the rate of positive lymph nodes (Table 2).

In the multivariate Cox proportional hazards model, histological subtype, AJCC stage, receipt of radiation therapy, and LNR were independently associated with OS after adjusting for sex, age, year of diagnosis, race, histology, AJCC stage, surgery, radiation, chemotherapy, and LNR.

**Figure 2:**
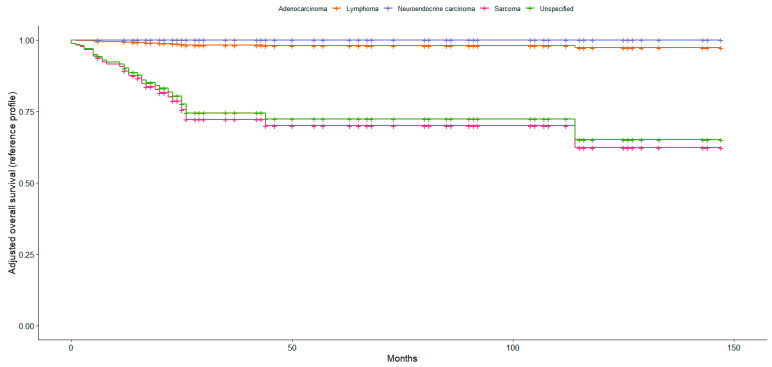
Adjusted survival curves by histologic subtype based on the Cox proportional hazards model, with adenocarcinoma specified as the reference category.

Compared with adenocarcinoma, patients with lymphoma had a significantly lower risk of death (HR 0.06, 95% CI 0.006–0.523, *p* = 0.011), while no statistically significant differences in OS were observed for neuroendocrine carcinoma, sarcoma, or unspecified histology. As illustrated in Figure 2, survival curves differed markedly by histological subtype. Adenocarcinoma, used as the reference category, was associated with the poorest survival, with other histological subtypes demonstrating more favourable survival trajectories over time.

**Figure 3:**
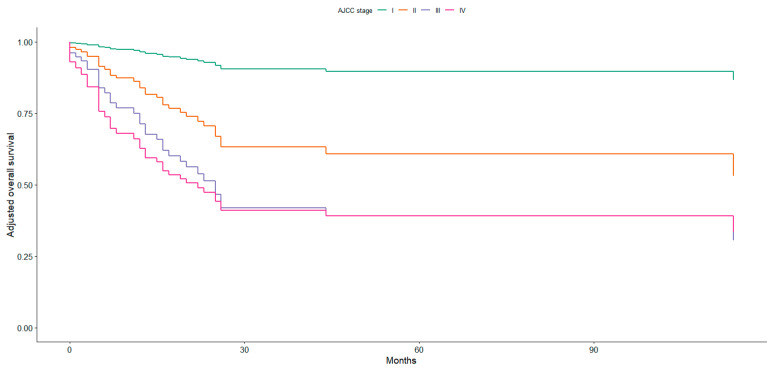
Adjusted survival curves by AJCC stage, based on Cox regression model.

Advanced AJCC stage was strongly associated with worse prognosis, with HRs of 5.13 (95% CI 1.20–21.9, *p* = 0.028) for stage II and 10.1 (95% CI 2.27–45.2, *p* = 0.002) for stage III compared with stage I; the association for stage IV did not reach statistical significance (HR 4.12, 95% CI 0.98–17.3, *p* = 0.053). Figure 3 demonstrates clear and progressive separation of survival curves according to AJCC stage, with higher stages associated with markedly reduced survival probabilities. The steep decline observed in stage III and IV disease highlights the strong prognostic impact of advanced tumour burden at diagnosis.

Radiation therapy was associated with improved survival in the multivariable model (HR 0.31, 95% CI 0.10–0.92, *p* = 0.035); however, this finding should be interpreted with caution given the lack of significance in univariate analysis. Higher LNR was associated with significantly increased mortality risk (HR 11.2, 95% CI 2.28–55.1, *p* = 0.003). No significant associations with overall survival were found for sex, age group, year of diagnosis, race, surgery, or chemotherapy.

Although 11 deaths occurred among patients with sarcoma and 6 among those with neuroendocrine carcinoma, model instability and potential collinearity in complete-case analyses led to exclusion or unreliable estimates for these subtypes in the final model. A complete overview of both unadjusted and adjusted estimates from the univariate and multivariable Cox regression analyses is provided in Table 3.

### 3.3. Sensitivity Analysis

In the multivariate Cox regression analysis, the adenocarcinoma histological subtype was stratified into signet ring cell carcinoma and other adenocarcinomas. Compared with signet ring cell carcinoma, other adenocarcinomas were associated with a significantly lower risk of mortality (HR 0.29, 95% CI 0.09–0.94, *p* = 0.038). All other risk factors remained consistent with the primary model, except for history of radiation, which lost borderline statistical significance (HR 0.34, 95% CI 0.11–1.01, *p* = 0.052).

Additional sensitivity analyses confirmed the robustness of these associations across multiple modelling approaches. In a complete-case multivariate Cox regression (n = 101), effect estimates closely paralleled the main analysis, with lymphoma (HR 0.06, 95% CI 0.01–0.52, *p* = 0.011), AJCC stage II (HR 5.13, 95% CI 1.20–21.9, *p* = 0.028) and III (HR 10.1, 95% CI 2.27–45.2, *p* = 0.002), and higher lymph node ratio (HR 11.2, 95% CI 2.28–55.1, *p* = 0.003) remaining independently associated with survival. In a second model treating lymph node ratio as a categorical variable (low, medium, high tertiles), the most recent diagnostic period (2013–2016) was associated with significantly lower mortality compared with 2004–2008 (HR 0.29, 95% CI 0.10–0.80, *p* = 0.017). Lymphoma remained strongly associated with improved survival (HR 0.04, 95% CI 0–0.41, *p* = 0.007), whereas advanced AJCC stage and medium (HR 6.37, 95% CI 1.85–21.9, *p* = 0.003) or high (HR 5.49, 95% CI 1.08–28.0, *p* = 0.040) lymph node ratio tertiles predicted significantly worse outcomes. Receipt of radiotherapy was again associated with reduced mortality risk (HR 0.17, 95% CI 0.05–0.56, *p* = 0.003). In the third model, excluding rare histological subtypes (NEC and unspecified), the pattern of associations was largely unchanged: lymphoma (HR 0.06, 95% CI 0.01–0.55, *p* = 0.0129), AJCC stage II (HR 4.88, 95% CI 1.12–21.3, *p* = 0.035) and III (HR 9.55, 95% CI 2.08–43.8, *p* = 0.0037), and higher lymph node ratio (HR 10.4, 95% CI 2.04–52.9, *p* = 0.0048) retained statistical significance, while radiation therapy maintained its association with improved survival (HR 0.32, 95% CI 0.11–0.94, *p* = 0.039).

## 4. Discussion

To the best of our knowledge, this is the first population-based cohort study to comprehensively describe the clinicopathologic features, treatment patterns, and survival outcomes of gastric malignancies in individuals aged ≤24 years using SEER data. In this large cohort of 324 patients diagnosed between 2004 and 2016, adenocarcinoma, particularly signet ring cell carcinoma, emerged as the most prevalent histological subtype, primarily affecting young adults. The majority of patients (59%) presented with stage IV disease, and overall prognosis was poor, with a median overall survival of 18 months and a 5-year survival rate of 49%. Survival outcomes varied significantly by histological subtype and tumour stage; patients diagnosed with lymphomas, sarcomas, or neuroendocrine carcinomas exhibited markedly superior OS compared to those with adenocarcinoma or signet ring cell carcinoma. In multivariable analysis, advanced AJCC stage and a higher lymph node ratio were independently associated with increased mortality. These findings underscore the aggressive nature of gastric adenocarcinomas in young patients and emphasise the prognostic relevance of histological subtype, disease stage at presentation, and lymph nodal involvement in this rare population.

The observed association between chemotherapy and worse survival likely reflects confounding by indication, as chemotherapy is preferentially administered to patients with advanced or aggressive disease. Similarly, the apparent survival benefit associated with radiotherapy in multivariable analysis should be interpreted cautiously, given the lack of significance in univariate analysis, small sample size, and absence of detailed treatment information in SEER. Importantly, the SEER database does not provide information on the timing, intent, or sequencing of chemotherapy, nor whether it was administered in combination with surgery. As such, it is not possible to distinguish between neoadjuvant, adjuvant, or palliative chemotherapy. This limitation further constrains the interpretation of treatment-related associations. Additionally, the observed survival differences between patients aged ≤18 years and those aged 19–24 years may reflect biological and clinical transitions across the paediatric–young adult continuum, including differences in tumour biology, treatment approaches, and healthcare delivery systems. Importantly, the inclusion of heterogeneous histological subtypes reflects the spectrum of primary gastric malignancies in this age group rather than a single disease entity. These tumour types differ substantially in biological behaviour, therapeutic strategies, and prognosis. Therefore, comparisons across histological subtypes should be interpreted as descriptive epidemiological observations rather than direct guidance for treatment decisions. While adenocarcinoma represents the most clinically relevant subtype, particularly in young adults, the present analysis was not designed to provide histology-specific therapeutic recommendations

Our findings align with those of a prior study of 144 patients from the National Cancer Data Base (NCDB), which also investigated paediatric and young adult gastric adenocarcinoma. Both studies focused on individuals aged ≤24 years and reported similar clinicopathologic patterns, including a predominance of adenocarcinoma and signet ring cell carcinoma, frequent presentation with advanced-stage disease, and poor long-term survival [10]. In that NCDB study, approximately 54% had metastatic disease at diagnosis, and the 5-year OS was 30%, compared to 49% in our SEER-based analysis. Surgical resection was more commonly performed in patients with non-metastatic disease in both studies, although fewer patients overall underwent surgery in the currently reported cohort (39% vs. 66% in NCDB) [10]. Notably, our study identified AJCC stage and lymph node ratio as independent predictors of survival, while the NCDB study emphasised the survival benefit of surgical resection and early-stage presentation. A key distinction lies in the database characteristics: SEER offers population-based coverage with broader epidemiologic insights, whereas NCDB is hospital-based and provides more detailed treatment data but lacks long-term survival follow-up [10]. Although surgical resection was significantly associated with improved survival in univariate analysis, this effect did not persist in the multivariable model. This likely reflects confounding by indication, as patients selected for surgery may have had more favourable disease characteristics, including earlier stage and resectable histology. Thus, the lack of statistical significance should be interpreted with caution and does not negate the potential benefit of surgery in appropriately selected patients. Similar to our study, the NCDB analysis did not find a statistically significant difference in OS according to age [10]. Collectively, our findings are broadly consistent with studies from Europe, Latin America, and Asia, which similarly report a predominance of diffuse-type and signet ring cell histology, advanced stage at diagnosis, and poor survival in younger patients. However, regional differences in incidence, ethnicity, and access to care may contribute to variability in outcomes.

The incidence of paediatric gastric adenocarcinoma is exceedingly rare in both Eastern and Western populations, with limited cases reported across regions. In a study from Japan, a nationwide survey identified 80 cases of gastric cancer in children and adolescents (<20 years) [11], mostly over 10 years old, with no sex differences, often presenting with abdominal pain and undifferentiated carcinoma. A notable family history of cancer (36%) and a link to *Helicobacter pylori* infection (2/3 tested positive) were also observed [11]. Analogously, in a multicenter study from Korea (1995–2016) involving 80 paediatric patients (<18 years) with adenocarcinoma, 15 cases (18.8%) were gastric adenocarcinomas [12]. The median age at diagnosis was 16 years (range, 12–17), with a male-to-female ratio of 1:1.14. Notably, 40% of patients had a family history of cancer, with three specifically linked to gastric cancer [12]. In a cohort from the Bavarian Cancer Registry (2002–2014) including 290 children aged 0–17 years with very rare paediatric tumours [13], predominantly adolescents (88.3% aged 10–17), no specific cases of gastric carcinoma were identified, though nine cases of colon and rectum carcinomas were reported, indicating the extreme rarity of gastric carcinoma in this paediatric population [13].

Paediatric gastric adenocarcinoma remains an exceptionally rare and aggressive malignancy, with limited data to inform clinical management [12,14]. In the largest Western single-centre series to date, Subbiah et al. [14] evaluated 13 patients with gastric adenocarcinoma ≤ 21 years. The median age at diagnosis was 18 years, and a striking 92% had poorly differentiated or signet ring cell histology. Most patients (85%) presented with unresectable or metastatic disease, and the overall prognosis was dismal, with a median survival of 1.5 years, similar to our study, despite chemotherapy and, in select cases, surgical resection [14]. Similarly, in a Korean multicentre study spanning 1995–2016, Yang et al. identified 15 cases of gastric adenocarcinoma [12]. The median age at diagnosis was 16 years (range: 12–17), with an even male-to-female distribution. Notably, 40% had a family history of cancer, and over half were diagnosed at stage III or IV. The 5-year OS was approximately 58%, though precise histology-specific outcomes were not detailed. Both studies emphasise the frequent late-stage diagnosis, predominance of aggressive histological subtypes, and overall poor outcomes associated with paediatric gastric adenocarcinoma, underscoring the need for heightened clinical awareness and dedicated research efforts in this vulnerable population [12,14].

Our observation that patients diagnosed in 2013–2016 had markedly lower mortality compared with those diagnosed in 2004–2008 may reflect broader improvements in cancer care for adolescents and young adults over the past decade. During this period, several factors likely contributed to better outcomes, including enhanced diagnostic imaging, more accurate histopathologic classification, and refinements in staging that facilitate earlier and more tailored interventions [15]. Advances in surgical techniques, perioperative management, and supportive care may have reduced treatment-related morbidity and improved postoperative recovery. In addition, wider adoption of multimodal treatment strategies—combining surgery, chemotherapy, radiotherapy, and, in selected histological types, targeted approaches—has expanded the therapeutic armamentarium for malignancies in young patients [15]. Notably, our findings on LNR align with growing evidence across paediatric malignancies—including differentiated thyroid cancer [16], papillary thyroid cancer [17], and Wilms tumour [18]—demonstrating its robust prognostic significance for recurrence, persistence, and survival. Studies in these settings have shown that higher LNR thresholds are associated with two- to fivefold increases in adverse outcomes, underscoring the biologic relevance of nodal burden beyond simple nodal positivity. These developments, together with greater access to specialised cancer centres, may underlie the survival advantage observed in the most recent diagnostic era in our cohort [15].

In line with prior SEER-based research on gastrointestinal malignancies in paediatric populations, recent findings from a large cohort of colorectal adenocarcinomas in patients under 18 years of age also highlight key parallels and distinctions [19]. Both analyses underscore the predominance of advanced-stage disease at diagnosis: stage IV tumours were present in 59% of our gastric carcinoma cohort and in 37.1% of paediatric colorectal cases. Histologic subtypes associated with aggressive behaviour were similarly frequent, with signet ring cell carcinoma comprising 19% of colorectal cases and emerging as an independent predictor of poor survival, consistent with its adverse prognostic role in our gastric cancer dataset. Despite differences in tumour location and age distribution—colorectal cancers more commonly involved the colon, while gastric tumours often arose proximally and in older adolescents or young adults—both studies converge on the prognostic significance of disease stage and histological subtype. Moreover, our analysis uniquely identified LNR and age as survival-associated factors. Methodologically, both studies share the inherent limitations of registry-based retrospective designs, including incomplete treatment data and lack of molecular profiling, reinforcing the necessity for prospective paediatric-specific oncologic registries to inform clinical practice.

Our study has several limitations, primarily related to its retrospective design and the use of registry-based data. The SEER database, while comprehensive, is subject to potential misclassification and reporting biases, as data are abstracted across multiple centres. A considerable proportion of cases had missing data, particularly for metastatic sites, tumour grade, and lymph node involvement, which limited the completeness of certain analyses and precluded the inclusion of these variables in multivariable models. The substantial proportion of missing data for key variables such as metastatic status and tumour grade may introduce selection bias and limit the internal validity of certain comparisons. To mitigate potential bias, variables with substantial missingness (exceeding 50%) and/or limited clinical relevance in this specific population—such as anatomical site, histological grade, and metastatic status (bone, liver, lung)—were excluded from survival analyses. These variables were analysed descriptively and, where appropriate, in relation to overall survival, with all available data transparently reported. Furthermore, sensitivity analyses, including complete-case modelling, yielded consistent results, supporting the robustness of the primary findings despite these limitations. Moreover, despite multivariable and sensitivity analyses being performed to account for confounding and to test the robustness of our findings, the imbalance in age distribution (with two-thirds of patients aged 20–24 years) limits statistical power in the younger subgroups and may reduce the generalisability of subgroup-specific estimates. The predominance of White patients in the SEER cohort may limit the generalisability of our findings to more diverse populations. Although race was included as a covariate in the analyses, the SEER database provides only broad racial categories (White, Black, Other), without more detailed ethnic stratification, thereby limiting the assessment of finer ethnic disparities. Furthermore, the lack of granular geographic data within SEER precludes a comprehensive evaluation of geographic variation in disease presentation and outcomes. Additionally, SEER lacks granular data on clinical variables of interest, such as performance status, specific chemotherapy regimens, molecular or genetic profiles (e.g., CDH1, MSI status), and family history of cancer, which are highly relevant in young-onset gastric malignancies. The absence of these variables limits a more comprehensive assessment of tumour biology and treatment response. Moreover, patients with missing survival time were excluded, as this variable is essential for time-to-event analyses, which may introduce selection bias. Furthermore, important potential confounders—such as socioeconomic status, access to healthcare, type of treatment facility, and insurance status—are not consistently captured in the SEER database. The lack of these variables may result in residual confounding and should be considered when interpreting the findings. Furthermore, the relatively small number of cases in certain subgroups may have reduced the statistical power and stability of some estimates. This is reflected in the wide confidence intervals observed for several covariates and likely indicates limited precision rather than the absence of effect. In particular, the attenuation of statistical significance for stage IV disease in the multivariable model, despite a strong effect size, likely reflects sparse data and potential collinearity with related variables such as lymph node ratio.

Similarly, the observed association between radiotherapy and improved survival should be interpreted with caution. The lack of significance in univariate analysis raises the possibility of confounding by indication, as patients selected for radiotherapy may have differed systematically from those who did not receive it. In addition, the relatively small number of patients receiving radiotherapy may have contributed to model instability. The absence of detailed data on radiotherapy dosage, intent, and treatment protocols within the SEER database further limits causal interpretation. Although limited information on treatment sequencing was available for a small subset of patients, this was insufficient for more granular analysis. Overall, these findings should be interpreted with appropriate caution.

## 5. Conclusions

Our population-based analysis of gastric malignancies in patients ≤ 24 years highlights the predominance of aggressive histological subtypes, advanced-stage presentation, and poor survival, especially in adenocarcinoma. Younger age, lower AJCC stage, and lower LNR were independently associated with improved OS. These findings reinforce the aggressive nature of early-onset gastric cancer and the need for earlier diagnosis and age-adapted therapeutic strategies. Future prospective studies with molecular data are essential to guide prognostic stratification in this rare population.

## Figures and Tables

**Table 1:** Baseline demographic, clinical, and tumour characteristics of the cohort (n = 324).

Variable	N (%) or Mean (SD)
**Age at diagnosis (years)**	20.1 (4.3)
**Sex, female**	162 (50%)
**Age category**	
*0–14 years*	34 (11%)
*15–19 years*	72 (22%)
*20–24 years*	218 (67%)
**Year of diagnosis**	
*2004–2008*	113 (35%)
*2009–2012*	101 (31%)
*2013–2016*	110 (34%)
**Race**	
*White*	228 (70%)
*Black*	43 (14%)
*Other*	52 (16%)
**Primary site**	
*Body*	71.0 (36)
*Antrum*	53.0 (27)
*Cardia*	47.0 (24)
**Histological subtype**	
*Adenocarcinoma*	168 (52%)
*Lymphoma*	56 (17%)
*Sarcoma*	59 (18%)
*NEC*	27 (9%)
*Unspecified*	14 (4%)
**Bone metastases**	9/147 (6%)
**Liver metastases**	25/149 (17%)
**Lung metastases**	16/149 (11%)
**AJCC stage group**	
*I*	53 (26%)
*II*	16 (8%)
*III*	13 (7%)
*IV*	117 (59%)
**Treatment modalities**	
*Surgery*	126 (39%)
*Radiation*	44 (14%)
*Chemotherapy*	186 (57%)

Percentages are calculated based on the number of patients with available data for each variable. NEC: neuroendocrine carcinoma; AJCC: American Joint Committee on Cancer.

**Table 2:** Distribution of tumour and treatment characteristics by age group.

Variable, n (%)	0–14 Years	15–19 Years	20–24 Years	*p*-Value
**Histological subtype**				**<0.01**
*Adenocarcinoma*	6 (18%)	32 (44%)	130 (60%)	
*Lymphoma*	13 (38%)	13 (18%)	30 (14%)	
*NEC*	4 (12%)	7 (10%)	16 (7%)	
*Sarcoma*	11 (32%)	16 (22%)	32 (15%)	
*Unspecified*	0 (0%)	4 (6%)	10 (4%)	
**Bone metastases**	0 (0%)	2 (6%)	7 (7%)	0.57
**Liver metastases**	4 (29%)	5 (16%)	16 (16%)	0.46
**Lung metastases**	2 (13%)	2 (6%)	12 (12%)	0.64
**AJCC stage IV**	7 (44%)	24 (59%)	86 (61%)	0.88
**Surgery**	14 (41%)	34 (46%)	78 (37%)	0.23
**Radiation**	4 (12%)	7 (10%)	33 (11%)	0.48
**Chemotherapy**	18 (54%)	37 (52%)	131 (61%)	0.37

Percentages are calculated based on the number of patients with available data for each variable. Bold *p*-value denotes statistical significance.

**Table 3:** Hazard ratios (HRs) with 95% confidence intervals (CIs) and *p*-values from univariate and multivariable Cox regression models for overall survival.

Variable	HR (95% CI)	*p*-Value	Adjusted HR (95% CI)	*p*-Value
**Age group**				
*0–14 years*	reference			
*15–19 years*	1.95 (0.89–4.29)	0.099	3.81 (0.43–33.6)	0.228
*20–24 years*	3.16 (1.54–6.47)	**<0.01**	5.16 (0.59–45.10)	0.138
**Male sex**	1.28 (0.93–1.76)	0.13	1.73 (0.66–4.55)	0.266
**Year of diagnosis**				
*2004–2008*	reference			
*2009–2012*	1.01 (0.70–1.46)	0.971	1.16 (0.45–2.98)	0.756
*2013–2016*	0.80 (0.53–1.20)	0.281	0.48 (0.19–1.22)	0.124
**Race**				
*White*	reference			
*Black*	1.11 (0.70–1.75)	0.670	1.62 (0.46–5.75)	0.455
*Other*	1.47 (0.98–2.22)	0.063	1.28 (0.54–3.04)	0.571
**Histology**				
*Adenocarcinoma*	reference			
*Lymphoma*	0.14 (0.07–0.26)	**<0.01**	0.06 (0.01–0.52)	**0.011**
*NEC*	0.18 (0.08–0.40)	**<0.01**	N/A	
*Sarcoma*	0.14 (0.08–0.27)	**<0.01**	N/A	
*Unspecified*	0.89 (0.43–1.83)	0.747	0.91 (0.06–14.70)	0.944
**AJCC stage**				
**I**	reference			
**II**	3.08 (1.07–8.91)	**0.037**	5.13 (1.20–21.90)	**0.028**
**III**	8.00 (3.07–20.86)	**<0.01**	10.10 (2.27–45.20)	**0.002**
**IV**	13.75 (6.62–28.58)	**<0.01**	4.12 (0.98–17.30)	**0.053**
**Surgery (yes)**	0.32 (0.22–0.47)	**<0.01**	0.61 (0.11–3.29)	0.565
**Radiation (yes)**	1.05 (0.67–1.63)	0.838	0.31 (0.10–0.92)	**0.035**
**Chemotherapy (yes)**	2.23 (1.56–3.19)	**<0.01**	1.19 (0.32–4.39)	0.798
**Rate of positive LNs**	1.72 (0.90–3.28)	0.101	11.20 (2.28–55.10)	**0.003**

N/A values reflect model convergence issues due to sparse data. Bold *p*-value denotes statistical significance; LNs: Lymph Nodes.

## Data Availability

The data presented in this study were derived from the Surveillance, Epidemiology, and End Results (SEER) database, which is a publicly available, de-identified resource maintained by the U.S. National Cancer Institute. Access to SEER data is available through SEER*Stat (https://seer.cancer.gov/) (accessed on 20 March 2026) upon registration and compliance with SEER data-use agreements.

## References

[1] Siegel R.L., Giaquinto A.N., Jemal A. (2024). Cancer statistics, 2024. CA Cancer J. Clin..

[2] Li J. (2020). Gastric Cancer in Young Adults: A Different Clinical Entity from Carcinogenesis to Prognosis. Gastroenterol. Res. Pract..

[3] Li J., Kuang X.H., Zhang Y., Hu D.M., Liu K. (2022). Global burden of gastric cancer in adolescents and young adults: Estimates from GLOBOCAN 2020. Public Health.

[4] Wu S.L., Zhang Y., Fu Y., Li J., Wang J.S. (2022). Gastric cancer incidence, mortality and burden in adolescents and young adults: A time-trend analysis and comparison among China, South Korea, Japan and the USA. BMJ Open.

[5] Attard T.M., Omar U., Glynn E.F., Stoecklein N., St Peter S.D., Thomson M.A. (2023). Gastric cancer in the pediatric population, a multicenter cross-sectional analysis of presentation and coexisting comorbidities. J. Cancer Res. Clin. Oncol..

[6] Santoro R., Carboni F., Lepiane P., Ettorre G.M., Santoro E. (2007). Clinicopathological features and prognosis of gastric cancer in young European adults. Br. J. Surg..

[7] Calderillo-Ruíz G., Díaz-Romero M.C., Carbajal-López B., Herrera-Martínez M., Ruiz-García E., Leon-Takahashi A.M., López-Basave H.N., Meneses-García A., Herrera-Gomez Á. (2023). Latin American young patients with gastric adenocarcinoma: Worst prognosis and outcomes. J. Gastrointest. Oncol..

[8] De B., Rhome R., Jairam V., Özbek U., Holcombe R.F., Buckstein M., Ang C. (2018). Gastric adenocarcinoma in young adult patients: Patterns of care and survival in the United States. Gastric Cancer Off. J. Int. Gastric Cancer Assoc. Jpn. Gastric Cancer Assoc..

[9] Heinze G., Wallisch C., Dunkler D. (2018). Variable selection—A review and recommendations for the practicing statistician. Biom. J. Biom. Z..

[10] Tessler R.A., Dellinger M., Richards M.K., Goldin A.B., Beierle E.A., Doski J.J., Goldfarb M., Langer M., Nuchtern J.G., Raval M.V. (2019). Pediatric gastric adenocarcinoma: A National Cancer Data Base review. J. Pediatr. Surg..

[11] Okuda M., Nomura K., Kato M., Lin Y., Mabe K., Miyamoto R., Okumura A., Kikuchi S. (2019). Gastric cancer in children and adolescents in Japan. Pediatr. Int. Off. J. Jpn. Pediatr. Soc..

[12] Yang H.B., Namgoong J.M., Kim K.H., Kim D.Y., Park J., Shin H.B., Youn J.K., Lee S., Lee J.W., Jung S.E. (2020). Pediatric Adenocarcinoma in Korea: A Multicenter Study. Cancer Res. Treat..

[13] Achajew A., Brecht I.B., Radespiel-Tröger M., Meyer M., Metzler M., Bremensdorfer C., Spix C., Erdmann F., Schneider D.T., Abele M. (2022). Rare pediatric tumors in Germany—Not as rare as expected: A study based on data from the Bavarian Cancer Registry and the German Childhood Cancer Registry. Eur. J. Pediatr..

[14] Subbiah V., Varadhachary G., Herzog C.E., Huh W.W. (2011). Gastric adenocarcinoma in children and adolescents. Pediatr. Blood Cancer.

[15] Ohlsen T.J.D., Martos M.R., Hawkins D.S. (2024). Recent advances in the treatment of childhood cancers. Curr. Opin. Pediatr..

[16] Qiu C., Wu S., Li J. (2024). Central lymph node ratio is an important recurrence prognostic factor for pediatric differentiated thyroid cancer. Front. Endocrinol..

[17] Xu Y., Wang Y., Zhang X., Huang R., Tian R., Liu B. (2021). Prognostic value of lymph node ratio in children and adolescents with papillary thyroid cancer. Clin. Endocrinol..

[18] Ziogas I.A., Khomiak A., Olson K.E., Moris D.P., Robbins A.J., Stevens J., Acker S.N., Roach J.P., Corkum K.S., Cost N.G. (2025). The Impact of Lymph Node Ratio for Children with Wilms Tumors: A National Cancer Database Analysis. Cancers.

[19] Emile S.H., Horesh N., Garoufalia Z., Gefen R., Dourado J., Wexner S.D. (2025). A SEER Registry-Based Analysis of Pediatric Colorectal Adenocarcinomas. JAMA Oncol..

